# Enhancement of Quality Factors in a 6.5 GHz Resonator Using Mo/SiC Composite Microstructures

**DOI:** 10.3390/mi16050529

**Published:** 2025-04-29

**Authors:** Binghui Lin, Yupeng Zheng, Haiyang Li, Yuqi Ren, Tingting Yang, Zekai Wang, Yao Cai, Qinwen Xu, Chengliang Sun

**Affiliations:** 1 Hubei Key Laboratory of Electronic Manufacturing and Packaging Integration, The Institute of Technological Sciences, Wuhan University, Wuhan 430072, China; cyrus0108@whu.edu.cn (B.L.); zhengyupeng@whu.edu.cn (Y.Z.); lhy2022@whu.edu.cn (H.L.); renyuqi@whu.edu.cn (Y.R.); tingtingyang@whu.edu.cn (T.Y.); zekaiwang@whu.edu.cn (Z.W.); caiyao999@whu.edu.cn (Y.C.); 2 Wuhan Institute of Quantum Technology, Wuhan University, Wuhan 430072, China

**Keywords:** FBAR, SiCOI substrate, microstructure, quality factor

## Abstract

This study addresses the critical challenge of lateral acoustic wave energy leakage in high-frequency film bulk acoustic resonators (FBARs) and elucidates the reflection mechanism of acoustic waves at acoustic reflection boundaries. Based on the theory of acoustic impedance mismatch, a novel Mo/SiC composite microstructure is designed to strategically establish multiple acoustic reflection boundaries along the lateral acoustic wave leakage paths. Finite element simulations reveal that SiC microstructures effectively suppress vibration amplitudes in non-resonant regions, thereby preventing acoustic wave leakage. By integrating Mo and SiC microstructures, the proposed composite structure significantly enhances the resonator’s acoustic confinement and energy retention capabilities. A resonator incorporating this Mo/SiC composite microstructure is fabricated, achieving a series resonance frequency of 6.488 GHz and a remarkable quality factor (Q) of 310. This represents a substantial 51.2% improvement in Q compared to the basic FBAR, confirming the effectiveness of the proposed design in mitigating lateral acoustic wave leakage and enhancing resonator performance for high-frequency, low-loss applications. This work offers valuable insights into the design of next-generation RF resonators for advanced wireless communication systems.

## 1. Introduction

With the rapid evolution of wireless communication technology, the global deployment of fifth-generation (5G) mobile networks is accelerating, while research efforts toward sixth-generation (6G) technology are intensifying [1,2]. The increasing demand for high-speed, low-latency communication necessitates the utilization of higher frequency bands, thereby driving the need for high-performance, low-loss RF devices [3,4]. In this context, FBARs play a crucial role in RF filtering applications due to their high electromechanical coupling efficiency and compact form factor. However, as operating frequencies continue to rise, conventional FBARs suffer from severe intrinsic losses in piezoelectric film and lateral acoustic wave leakage, leading to significant energy dissipation and reduced quality factors, which ultimately degrade device performance.

To address this challenge, researchers have focused on optimizing the quality of piezoelectric thin films and developing novel resonator architectures to suppress energy losses. However, the inherent limitations of piezoelectric film growth on non-single-crystalline substrates restrict the extent of quality improvement achievable through growth optimization and thermal treatment [5,6,7,8,9,10,11]. Moreover, while considerable efforts have been devoted to suppressing acoustic wave leakage along the longitudinal direction, relatively little attention has been given to mitigating lateral acoustic wave energy dissipation [12,13,14,15,16,17,18,19]. Few studies have reported the implementation of Bragg reflectors or frame structures in the lateral direction of resonators [20,21]. Their work provides a good guide for suppressing lateral acoustic wave energy leakage. However, these structures typically incorporate top electrode materials, which prevent the formation of a well-defined acoustic impedance mismatch boundary on the surface. The issue becomes increasingly critical at high frequencies, where reduced piezoelectric film thicknesses exacerbate intrinsic losses and shorter acoustic wavelengths enhance wave transmission, leading to substantial energy leakage from the resonator surface and edges.

In this work, we propose and investigate an innovative Mo/SiC composite microstructured FBAR that effectively suppresses lateral acoustic wave leakage by establishing multiple acoustic reflection boundaries. Through a combination of theoretical analysis, finite element simulations, and experimental validation, we demonstrate the ability of this microstructure to enhance acoustic confinement and improve resonator performance. Based on high-quality Sc_0.2_Al_0.8_N grown on SiCOI substrates, the fabrication process is refined using an optimized thin-film bonding and transfer technique, ensuring high structural integrity and device reliability. The experimental results confirm that the Mo/SiC composite microstructured FBAR achieves a significant Q factor enhancement of 51.2%, paving the way for the next generation of high-frequency acoustic devices.

## 2. Structural Design and Simulation

**Figure 1:**
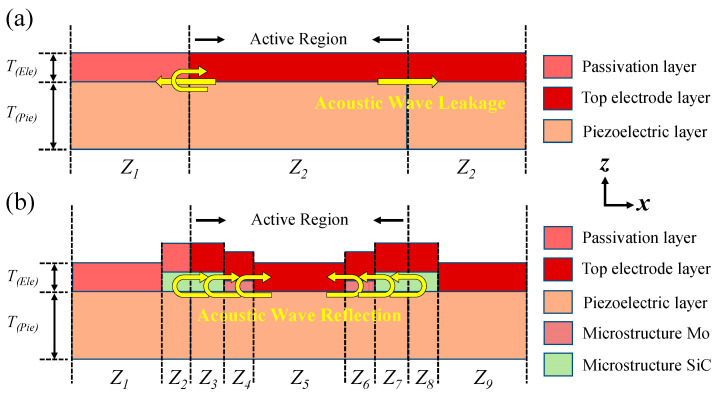
Schematic diagram of the acoustic boundary. (**a**) The basic FBAR has lateral energy leakage due to the lack of an acoustic reflection boundary. (**b**) The FBAR configured with Mo/SiC composite microstructures constructs an acoustic reflection boundary to suppress lateral energy leakage.

FBARs typically consist of a sandwich structure comprising a top electrode layer, a piezoelectric layer, and a bottom electrode layer. When a voltage is applied across the electrodes, the d33 piezoelectric coefficient of the piezoelectric film excites the fundamental mode (thickness-extensional mode), while the d31 coefficient induces parasitic modes (lateral modes). Additionally, in practical applications, the metal routing paths of the top and bottom electrodes are not perfectly aligned along the z-axis. This misalignment at the resonator’s edge results in lateral electric field components, which exacerbate lateral vibrations in the piezoelectric film. As illustrated in Figure 1a, these lateral vibrations generate acoustic waves that propagate along the x-axis within the film. While some waves are reflected at the interface between the top electrode layer and the passivation layer, a portion of the acoustic energy still escapes from the resonator region.

Acoustic waves undergo reflection at acoustic boundaries where impedance mismatches occur. By constructing multiple acoustic boundaries, it is possible to maximize acoustic energy retention. To explore the feasibility of local acoustic impedance modulation within the resonator structure, two candidate materials—molybdenum (Mo) and silicon carbide (SiC)—were evaluated based on their acoustic properties. Table 1 summarizes the density, longitudinal acoustic velocity, and calculated acoustic impedance of these materials. Mo exhibits a high acoustic impedance due to its high density and velocity, whereas SiC shows a lower impedance, resulting from its relatively lower density but much higher velocity. These significant differences in acoustic impedance compared to that of the surrounding materials suggest that both Mo and SiC can serve as effective localized reflectors or impedance modulation regions. Moreover, the superior mechanical strength of SiC and the ease of thin-film deposition for Mo render them highly suitable for the design of acoustic wave reflection structures. When introduced in the lateral plane of the resonator, they are expected to generate impedance mismatch boundaries that partially reflect the laterally propagating acoustic waves, thereby suppressing lateral energy leakage and improving Q performance.

**Figure 2:**
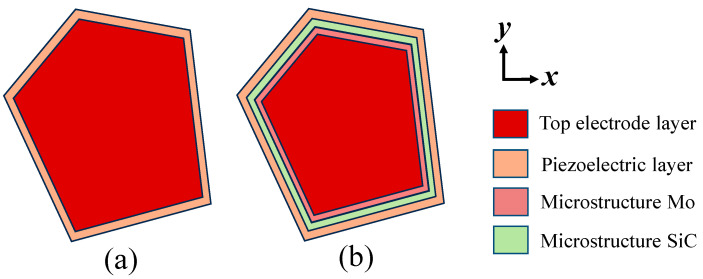
Schematic top view of (**a**) the basic FBAR and (**b**) the FBAR configured with Mo/SiC composite microstructures. The Mo/SiC composite microstructures are distributed around the boundary of the resonance region.

This study proposes a Mo/SiC composite microstructure, as illustrated in Figure 1b, which depicts the acoustic boundary configuration of an FBAR incorporating Mo and SiC microstructures. Region 1, covered by the passivation layer, exhibits an effective acoustic impedance determined by the density and Young’s modulus of both the passivation and piezoelectric layers. Regions 5 and 9, covered by the top electrode layer, have an effective acoustic impedance influenced by the density and Young’s modulus of both the top electrode and piezoelectric layers. Regions 4 and 6, covered by both the top electrode and Mo microstructure, have an effective acoustic impedance determined by the density and Young’s modulus of the top electrode, Mo microstructure, and piezoelectric layer. Similarly, regions 3, 7, and 8, covered by the top electrode and SiC microstructure, exhibit an effective acoustic impedance dependent on the top electrode, SiC microstructure, and piezoelectric layer. By introducing Mo and SiC microstructures within the active region, multiple acoustic reflection boundaries near the resonator’s edges are created. This configuration facilitates multiple reflections of laterally leaked acoustic waves before they escape the resonator region, effectively suppressing lateral acoustic leakage. Furthermore, the high crystalline quality of single-crystal SiC prevents excessive energy dissipation within the material. Figure 2 shows the schematic top view of the basic FBAR and the FBAR configured with Mo/SiC composite microstructures. The Mo/SiC composite microstructures are distributed around the boundary of the resonance region.

This paper utilizes COMSOL^®^ finite element simulation to optimize the design of high-frequency film bulk acoustic wave resonators. To validate this theoretical analysis, simulations compare the performance of two FBAR configurations: (1) a resonator without microstructures and (2) a resonator with microstructures. Two-dimensional simulation models are performed to investigate the resonance frequency and the propagation mode of the FBAR. The substrate, cavity, and sandwich stack (including the top electrode layer, piezoelectric layer, and bottom electrode layer) are constructed in the geometry module. The relative position relationship of each part and the width and thickness of each film layer are confirmed. The width of the two-dimensional model of the film bulk acoustic wave resonator is 200 μm, the thickness of the substrate is 10 μm, the width and depth of the cavity are 100 μm and 1.5 μm, respectively, the thicknesses of the top electrode layer and the bottom electrode layer are 65 nm each, and the thickness of the piezoelectric layer is 270 nm. The design parameters of the FBAR are shown in Table 2.

**Figure 3:**
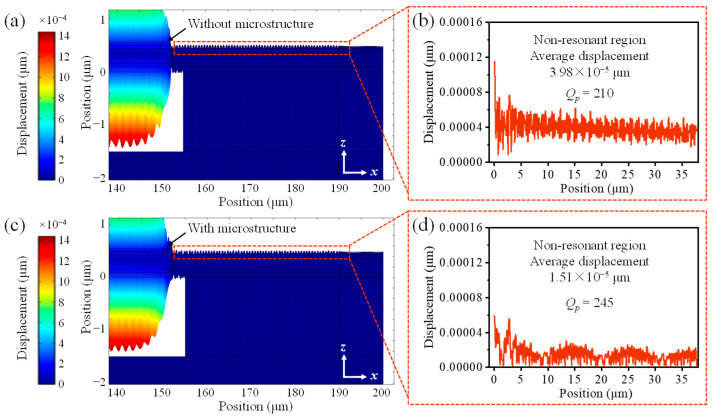
Mode shape and displacement curve of the resonator (**a**,**b**) without and (**c**,**d**) with SiC microstructures.

As shown in the results in Figure 3, at the resonant frequency, the vibration of the film is mainly concentrated in the resonant region above the cavity, forming a significant main resonant mode, and the acoustic wave energy is also concentrated in the resonant region. The basic FBAR shows that acoustic energy leaks continuously from the resonator to the non-resonant region, resulting in an average vibration displacement of approximately 3.98 × 10^−5^ μm. However, when 50-nanometer-thick SiC microstructures are introduced, the average vibration displacement in the non-resonant region is reduced to 1.51 × 10^−5^ μm, demonstrating significant suppression of lateral acoustic leakage.

The material properties and dimensions of the microstructures are critical for suppressing lateral acoustic energy leakage. The key acoustic properties (acoustic impedance) of the Mo and SiC materials used in the simulation are listed in Table 1. To effectively reflect and interfere with lateral acoustic waves, the width of the microstructure should be designed as an odd multiple of a quarter wavelength of the corresponding lateral wave mode at the resonance frequency [21]:

(1) $$width=(2m+1)\frac{\lambda }{4}$$

where m is an ideally positive integer.

According to the simulated dispersion characteristics of the FBAR configuration, the wavelength of the lateral S_0_ mode is 840 nm, and that of the A_0_ mode is 810 nm. Therefore, the width of the microstructures should be designed as an odd multiple of 202.5 nm or 210 nm to achieve maximum constructive interference and reflection of lateral acoustic waves, thereby effectively suppressing energy leakage.

**Figure 4:**
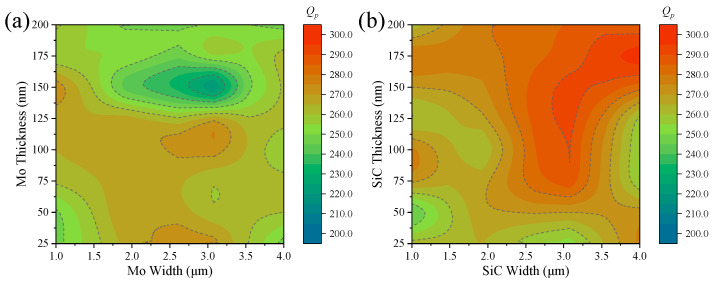
Trend in Qp variation in resonators with (**a**) Mo or (**b**) SiC microstructure dimensions.

By incorporating Mo microstructure within the resonant region, local material thickness can be modified, thereby adjusting the effective density and Young’s modulus to establish acoustic reflection boundaries. This section investigates the effects of Mo microstructure width and thickness on the performance of the resonator. As illustrated in Figure 4a, the quality factor (Qp) of the resonator is analyzed under various Mo microstructure configurations, with widths of 1 μm, 2 μm, 3 μm, and 4 μm, and thicknesses ranging from 25 nm to 200 nm in increments of 25 nm. The results indicate that the introduction of Mo microstructures leads to varying degrees of Qp enhancement. Overall, the Qp values of the resonator incorporating Mo microstructures range from 218 to 273, representing an improvement of 3.8% to 30% compared to the basic resonator (Qp = 210). This demonstrates the effectiveness of Mo microstructures in mitigating acoustic energy leakage in thin-film bulk acoustic resonators (FBARs). Furthermore, the Qp variation exhibits a nonlinear dependence on the width and thickness of the Mo microstructures. Excessively narrow Mo microstructures may lack sufficient mechanical strength, leading to deformation during resonance and ineffective reflection of high-frequency acoustic waves. Conversely, overly thick Mo microstructures can introduce modal conversion and stress concentration, exacerbating energy leakage. The most favorable enhancement in Qp is observed when the Mo microstructure width is within the range of 1.5 μm to 3.5 μm and the thickness is below 125 nm.

By integrating SiC microstructures within the resonant region, local material composition can be altered, allowing for modulation of the effective density and Young’s modulus to create acoustic reflection boundaries. This section examines the impact of SiC microstructure width and thickness on resonator performance. As shown in Figure 4b, the Qp values of the resonator are evaluated under different SiC microstructure configurations, with widths of 1 μm, 2 μm, 3 μm, and 4 μm, and thicknesses varying from 25 nm to 200 nm in increments of 25 nm. The results reveal that the inclusion of SiC microstructures significantly enhances the Qp of the resonator. Specifically, the Qp values range from 245 to 297, corresponding to an improvement of 16.7% to 41.4% compared to the basic resonator (Qp = 210). This confirms the effectiveness of SiC microstructures in reducing acoustic energy leakage in FBARs. The relationship between Qp and SiC microstructure dimensions follows a nonlinear trend. Due to its high mechanical strength, a sufficiently thick SiC microstructure minimizes structural deformation, thereby effectively suppressing lateral acoustic wave leakage and confining more acoustic energy within the resonant region. The most significant enhancement in Qp is achieved when the SiC microstructure width is between 2.5 μm and 4 μm, with a thickness exceeding 125 nm.

However, a resonator incorporating microstructure exhibits significant spurious modes below the fundamental mode frequency. The frequency position of these spurious modes varies with the thickness of the microstructure. This phenomenon arises because when the microstructure is located within the resonant region, the layered thin-film structure in the thickness direction forms a local resonator. A portion of the electrical energy couples into localized standing waves, leading to resonance. Additionally, the increased thickness of the layered thin films in this region extends the acoustic wave propagation path, resulting in the appearance of spurious modes below the fundamental mode frequency. As the width of the microstructure increases, its proportion within the active region of the resonator grows, leading to an enhancement in the intensity of the spurious modes. Therefore, it is crucial to mitigate the impact of these spurious modes.

To minimize the impact of spurious modes on high-frequency communication bands, in addition to reducing the spurious mode frequency by increasing the film thickness, an alternative approach involves weakening the intensity of individual spurious modes through differential microstructures. As analyzed previously, the thickness of the microstructure affects the frequency position of the spurious modes, while its width influences their intensity. By dividing the microstructure into two sections with different thicknesses, it is possible to generate two spurious modes at distinct frequency positions, each with significantly reduced intensity. This design enhances the quality factor Qp of the resonator while ensuring that the spurious modes do not interfere with high-frequency communication bands.

**Figure 5:**
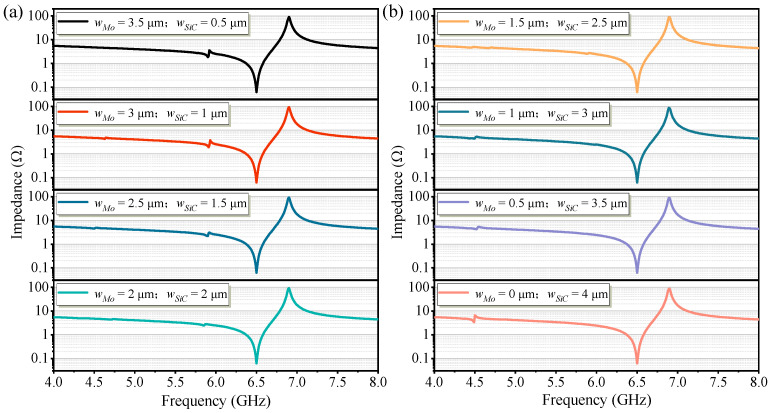
Simulated impedance curves of Mo/SiC composite microstructure resonators with different width ratios: (**a**) The width of Mo was set to 3.5 μm, 3 μm, 2.5 μm, and 2 μm, respectively. (**b**) The width of Mo was set to 1.5 μm, 1 μm, 0.5 μm, and 0 μm, respectively.

According to previous analysis, a Mo microstructure with a thickness of 35 nm or a SiC microstructure with a thickness of approximately 150 nm exhibits a noticeable enhancement in Qp. Based on this, the present work proposes a Mo/SiC composite microstructure, where a 35-nanometer-thick Mo layer and a 150-nanometer-thick SiC layer are combined. The microstructure within the resonant region is divided into two sections along the width direction, and the width ratio of these sections is optimized to suppress spurious mode vibrations. The composite Mo/SiC microstructure within the active region configures with a fixed total width of 4 μm. As shown in Figure 5, eight design variations were considered by adjusting the relative widths of Mo and SiC: the width of Mo was set to 3.5 μm, 3 μm, 2.5 μm, 2 μm, 1.5 μm, 1 μm, 0.5 μm, and 0 μm, respectively, corresponding to SiC widths of 0.5 μm, 1 μm, 1.5 μm, 2 μm, 2.5 μm, 3 μm, 3.5 μm, and 4 μm. By evaluating the intensity of spurious modes across these eight configurations, the optimal structure was selected to achieve enhanced Q for the fundamental mode while minimizing spurious mode interference. When the Mo microstructure width was 3.5 μm, a prominent spurious mode appeared near 5.9 GHz. Conversely, when the SiC microstructure width was 4 μm, a noticeable spurious mode emerged around 4.5 GHz. However, when the Mo microstructure width was set to 1.5 μm and the SiC microstructure width to 2.5 μm, no significant spurious modes were observed near 5.9 GHz or 4.5 GHz. These results preliminarily validate the feasibility of mitigating spurious modes by segmenting the microstructure within the resonant region and optimizing the width ratio of the two sections.

## 3. Thin Film Growth and Device Fabrication

Traditional FBAR fabrication relies on sacrificial SiO_2_ layers embedded within the substrate. However, due to their amorphous nature and high surface roughness, these layers create suboptimal templates for piezoelectric film growth, leading to degraded film quality and increased energy losses. To overcome these limitations, we deposit a 270-nanometer-thick Sc_0.2_Al_0.8_N film on a silicon-on-carbide (SiCOI) substrate.

**Figure 6:**
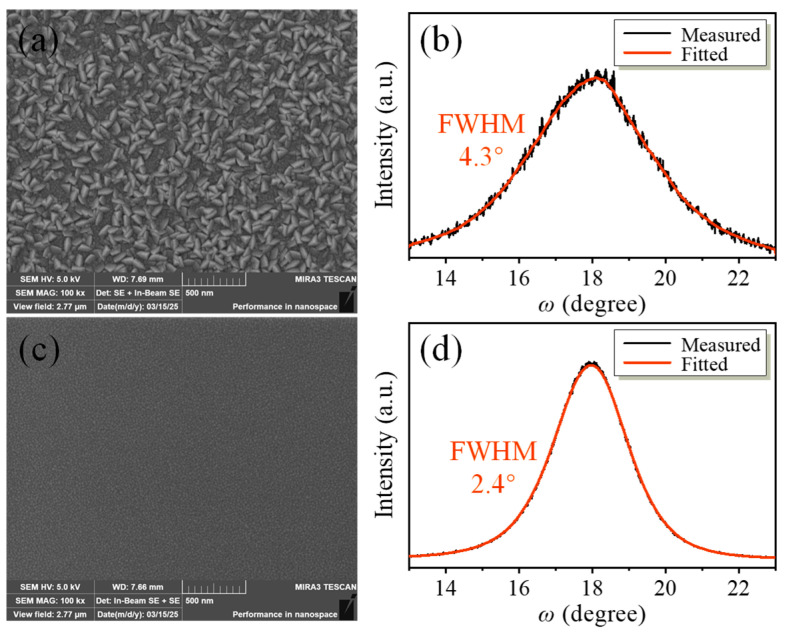
SEM images and rocking curves of 270-nanometer-thick Sc_0.2_Al_0.8_N films grown on (**a**,**b**) SiO_2_ film and (**c**,**d**) SiCOI substrate.

As shown in Figure 6, to assess the surface morphology and crystalline orientation of the deposited films, scanning electron microscope (SEM) and X-ray diffraction (XRD) measurements, including rocking curve scans, were conducted. Benefiting from the SiC film, which provides an excellent template for the growth of piezoelectric films, the Sc_0.2_Al_0.8_N film grown on the SiCOI substrate exhibits superior crystalline quality, reduced surface roughness, and a full-width at half maximum (FWHM) of 2.4° on the X-ray rocking curve, significantly lower than the 4.3° observed in conventional growth techniques. Moreover, no significant grain precipitation was observed on the film surface. This is very helpful in solving the severe intrinsic losses in piezoelectric film in high-frequency resonators.

**Figure 7:**
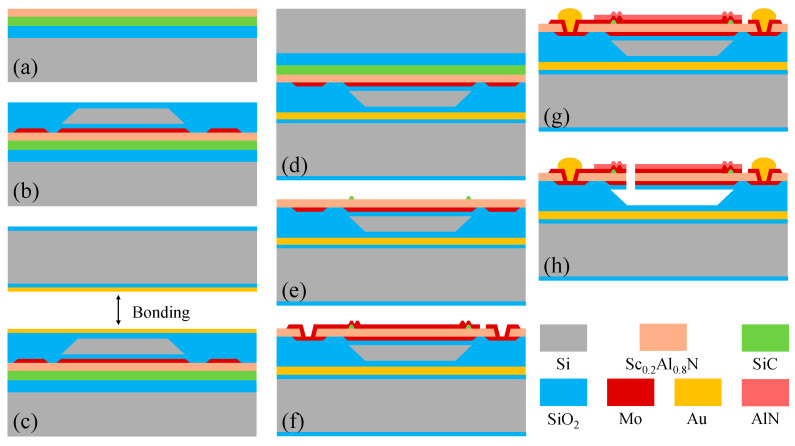
The main process step for the fabrication of FBAR. (**a**) Piezoelectric layer deposition. (**b**) Bottom electrode, sacrificial layer, and support layer fabrication. (**c**) Bonding material deposition. (**d**) Wafer bonding. (**e**) Si substrate and SiO_2_ film removal, and SiC microstructure fabrication. (**f**) Mo microstructure and top electrode fabrication. (**g**) Contact pad fabrication. (**h**) Release holes are etched, and a cavity is formed.

The FBAR is fabricated using an optimized thin-film bonding and transfer process. The detailed fabrication process flow of the proposed FBAR is illustrated in Figure 7. Firstly, a 270-nanometer-thick Sc_0.2_Al_0.8_N piezoelectric layer is deposited on the SiCOI substrate. A 65-nanometer-thick Mo is subsequently deposited and etched to form the bottom electrode. Then, a 100 nm-thick SiO_2_ is deposited to cover the wafer by plasma-enhanced chemical vapor deposition. The 1.4-micrometer-thick poly-Si layer is deposited and patterned to form the sacrificial layer. Next, a thick SiO_2_ support layer is deposited to fulfill the step height, and the wafer is flattened by using chemical mechanical polishing. After that, wafer bonding was performed using gold as the bonding medium between the two substrates. The Si substrate and SiO_2_ film are removed by wafer thinning and wet etching. In the next step, the SiC film is patterned and dry-etched to form a 150-nanometer-thick SiC microstructure. Subsequently, the piezoelectric layer is etched to expose the pad of the bottom electrode, followed by the deposition and etching of the 35-nanometer-thick Mo microstructure and the 65-nanometer-thick top electrode. After a 1-micrometer-thick Au film is deposited and patterned to define the contact pad, the 50-nanometer-thick passivation layer AlN covers the active region. At last, the release holes are etched, and the air cavity is released by xenon difluoride (XeF₂), thus finishing the fabrication of the proposed FBAR.

## 4. Measurement and Results Analysis

**Figure 8:**
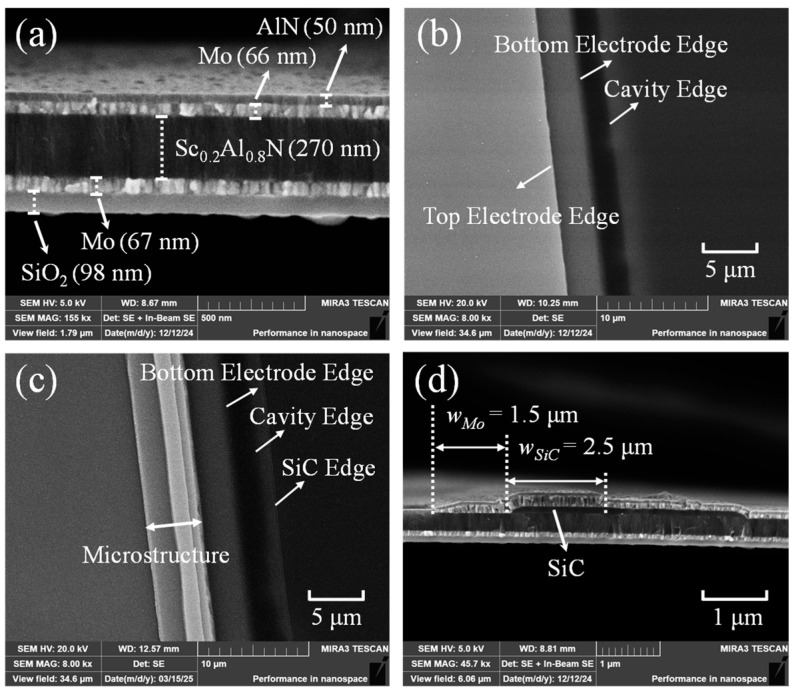
SEM images characterization: (**a**) The cross-sectional view of the basic FBAR. (**b**) Top view of the active region edge of the basic FBAR. (**c**) Top view of the Mo/SiC composite microstructure of the optimized FBAR. (**d**) The cross-sectional view of the Mo/SiC composite microstructure.

Figure 8a,b show the SEM cross-sectional view and top view of the basic FBAR. In the top-view magnified observation of the active region edge, the boundaries of the cavity, bottom electrode, and top electrode can be clearly identified. Figure 8c,d show the SEM top view and the cross-sectional view of the optimized FBAR. The Mo/SiC composite microstructures are arranged along the active region edge, with the first-stage Mo structure measuring 1.5 μm in width and the second-stage SiC structure measuring 2.5 μm in width.

The fabricated FBARs were measured using a radio frequency (RF) S-parameter measurement system. The system primarily consists of a semi-automatic RF probe station, a vector network analyzer, and GSG probes. The effective electromechanical coupling coefficient ($k_{eff}^{2}$) and quality factor (Q) can be calculated by the following formulas [22,23]:

(2) $$k_{eff}^{2}=\frac{\pi^{2}}{4}\cdot (\frac{f_{s}}{f_{p}})\cdot (\frac{f_{p}-f_{s}}{f_{p}})$$

(3) $$Q(f)=\frac{2\pi f\tau (f)|S_{11}|}{1-|S_{11}{|}^{2}}$$

where *τ*(*f*) is the group delay of *S*_11_.

**Figure 9:**
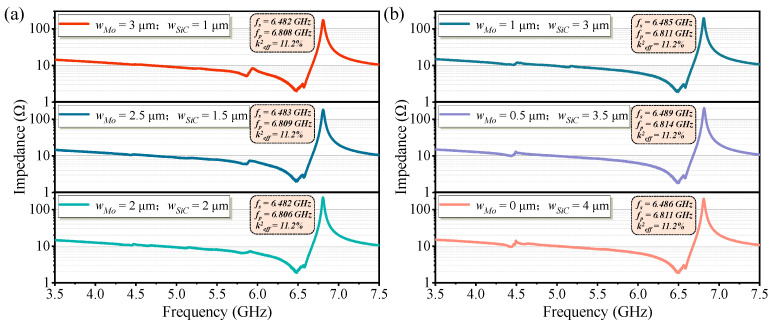
Measured impedance curves of Mo/SiC composite microstructure resonators with different width ratios: (**a**) The width of Mo was 3 μm, 2.5 μm, and 2 μm, respectively. (**b**) The width of Mo was 1 μm, 0.5 μm, and 0 μm, respectively.

Figure 9 shows the measured impedance curves of high-frequency FBARs with Mo/SiC composite microstructures of varying width ratios within the active region, aiming to analyze the variation of spurious modes under different configurations. As the width of Mo in the composite microstructure decreases, the spurious mode near 5.9 GHz gradually becomes suppressed. Correspondingly, as the width of SiC increases, the spurious mode near 4.5 GHz becomes more prominent. These experimental results are consistent with the simulation conclusions. To avoid the generation of spurious modes in the resonator, a balance between the widths of Mo and SiC in the composite microstructure is required.

**Figure 10:**
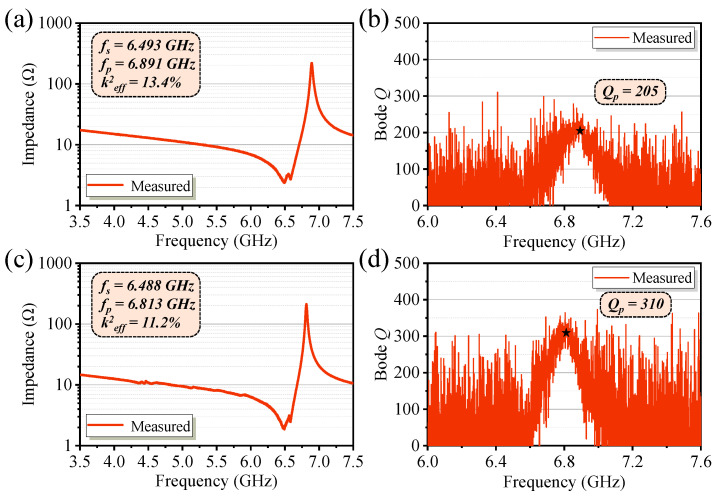
Measured impedance curves and Qp value of the resonator: (**a**,**b**) Basic resonator; (**c**,**d**) Optimized FBAR.

Figure 10 shows the measured impedance curves and Qp value of the basic resonator and the optimized FBAR. The basic FBAR exhibits a series resonance frequency (*fs*) of 6.493 GHz and a parallel resonance frequency (*fp*) of 6.891 GHz, corresponding to an effective electromechanical coupling coefficient ($k_{eff}^{2}$) of 13.4%. The quality factor at the parallel resonance frequency (Qp) is 205. The optimized FBAR (*w_Mo_* = 1.5 μm, *w_SiC_* = 2.5 μm) exhibits an *fs* of 6.488 GHz and an *fp* of 6.813 GHz. The Qp value reaches 310, and no significant spurious modes are observed. The Qp value of the optimized FBAR was improved by 51.2% compared to the basic FBAR. Impedance analysis reveals that the effective electromechanical coupling coefficient decreases from 13.4% in the basic FBAR to 11.2% in the optimized FBAR due to the introduction of additional boundary conditions. This is attributed to the presence of Mo/SiC composite microstructures within the active region, which couple a portion of the electrical energy into localized standing waves, resulting in the formation of spurious modes and a reduction in the electromechanical conversion efficiency of the fundamental mode.

The basic and optimized FBARs were characterized, and the Qp values of five devices from each type are summarized in Table 3. The mean and standard deviation were also calculated accordingly. The results indicate a significant improvement in Qp values for the optimized FBARs after structural optimization. Optimized microstructure dimensions prevent spurious modes, ensuring improved device performance. The quality factor (Qp) of the optimized resonator reaches 310, marking a 51.2% increase from the basic Qp of 205.

In summary, by introducing Mo/SiC composite microstructures within the active region to suppress lateral acoustic energy leakage, the Qp value of the resonator was successfully improved. Meanwhile, to address the spurious modes induced by microstructures in the active area, an optimized width configuration of the Mo/SiC composite structure was proposed, effectively eliminating the spurious responses. While existing studies focus on pushing FBARs toward higher frequencies, the Qp values of high-frequency FBARs are generally limited (typically below 300) due to intrinsic losses in the piezoelectric film and severe lateral acoustic energy leakage, as summarized in Table 4. In comparison, this work demonstrates a high-frequency, low-loss FBAR with a series resonance frequency of 6.488 GHz and a Qp value as high as 310, enabled by the growth of high-quality Sc₀.₂Al₀.₈N piezoelectric film on the SiC layer and the implementation of SiC-based composite microstructures.

## 5. Conclusions

This work successfully demonstrates a high-frequency, low-loss FBAR by suppressing lateral acoustic wave leakage using Mo/SiC microstructures. Through theoretical analysis, numerical simulations, and experimental validation, a high-frequency, low-loss FBAR with a quality factor of 310 and a series resonance frequency of 6.488 GHz was successfully demonstrated. By optimizing the piezoelectric film properties and refining the resonator structure, the dissipation of resonance energy at high frequencies was effectively mitigated, leading to a significant enhancement in the quality factor. The findings provide both theoretical foundations and experimental support for the advancement of FBAR technology in RF applications, facilitating the commercialization of high-frequency acoustic filters.

## Figures and Tables

**Table 1:** Main material properties of Mo and SiC.

Material	Density (kg/m^3^)	Velocity (m/s)	Acoustic Impedance (10^6^ kg/m^2^·s)	Mechanical Strength	Depositable
Mo	10,200	6260	63.9	Moderate	Yes
SiC	3200	12,000	38.4	High	Yes

**Table 2:** Design parameters of the resonator.

Function	Material	Thickness
Passivation layer	AlN	50 nm
Top electrode	Mo	65 nm
Piezoelectric layer	Sc_0.2_Al_0.8_N	270 nm
Bottom electrode	Mo	65 nm
Insulation layer	SiO_2_	100 nm
Microstructure 1	Mo	25 nm~200 nm
Microstructure 2	SiC	25 nm~200 nm

**Table 3:** The Qp values of five basic FBARs and five optimized FBARs, along with their mean and standard deviation.

Type	Device 1	Device 2	Device 3	Device 4	Device 5	Mean Value	Standard Deviation
Basic FBAR	201	201	202	202	205	202.2	1.64317
Optimized FBAR	302	304	309	309	310	306.8	3.56371

**Table 4:** Comparison with other works.

Piezoelectric Material and Thickness	Substrate	Frequency	Quality Factor	Reference
Sc_0.15_Al_0.85_N	Si	2.15 GHz	410	[24]
Sc_0.3_Al_0.7_N	SOI	2.93 GHz	210	[25]
Sc_0.2_Al_0.8_N	Si/SiO_2_	3.094 GHz	209	[26]
Sc_0.2_Al_0.8_N	Si/SiO_2_	3.39 GHz	193	[27]
Sc_0.2_Al_0.8_N	Si/SiO_2_	3.521 GHz	255.4	[28]
Sc_0.2_Al_0.8_N	Si/SiO_2_	4.001 GHz	202	[29]
Sc_0.2_Al_0.8_N	Si/SiO_2_	4.235 GHz	287.4	[30]
Sc_0.3_Al_0.7_N	Si	4.38 GHz	194	[31]
Sc_0.2_Al_0.8_N	Si	5.611 GHz	193	[32]
AlN	Si/SiO_2_	6.84 GHz	135	[33]
Sc_0.2_Al_0.8_N	SiC	6.488 GHz	310	This work

## Data Availability

The data presented in this study are available in this article.
